# Supplementary material for: Immunoglobulin Replacement Therapy is critical and cost-effective in increasing life expectancy and quality of life in patients suffering from Common Variable Immunodeficiency Disorders (CVID): A health-economic assessment
Source: PLoS One. 2021 Mar 4;16(3):e0247941. doi: 10.1371/journal.pone.0247941 (PMC7932530; doi:10.1371/journal.pone.0247941)
Supplement: S1 Table — (PDF) [file pone.0247941.s001.pdf]

S1 Table. Disutility values and utility calculation

Health state	Utilities	Disutility weight (D)[1]	U (= 1-D)
Infection			
	infection moderate	0.051	0.949
	infection severe	0.125	0.875
	post-infection	0.217	0.783
Autoimmunity	Crohn disease	0.221	0.779
	inflammatory bowel disease	0.062	0.938
	anemia mild	0.004	0.996
	anemia moderate	0.045	0.955
	anemia severe	0.118	0.882
	hypothyroidism	0.022	0.978
	thrombocytopenic purpura	0.167	0.833
Malignancy	cancer primary	0.265	0.735
	cancer advanced	0.358	0.642
Chronic lung disease	asthma controlled	0.020	0.980
	asthma severe	0.045	0.955
	COPD mild	0.025	0.975
	COPD moderate	0.284	0.716
	COPD severe	0.418	0.582

[1] Disutility values were derived from: National Institute for Public Health and the Environment. Ministry of Health, Welfare and Sport. Quantification and valuation of the human health impacts of chemicals based on quality and disability-adjusted life-years. Final Report. ECHA Framework Contract – QALYs and DALYs SR19, 2015.
