# Supplementary material for: Immunoglobulin Replacement Therapy is critical and cost-effective in increasing life expectancy and quality of life in patients suffering from Common Variable Immunodeficiency Disorders (CVID): A health-economic assessment
Source: PLoS One. 2021 Mar 4;16(3):e0247941. doi: 10.1371/journal.pone.0247941 (PMC7932530; doi:10.1371/journal.pone.0247941)
Supplement: S2 Table — (PDF) [file pone.0247941.s002.pdf]

S2 Table. Data sources for economic modeling and evaluation

**Health economic modeling**	
Source	*Description*
Liu et al. (2006) [1]	Health economic report, investigating the feasibility of health economic modeling in patients suffering from primary immunodeficiency disease. Proposal for Markov cohort model. Identified lack of reliable data on mortality, utility and costs.
**Cost elements**	
Source	*Description*
Sadeghi et al. (2015) [2]	Estimation of CVID cost drivers: mainly hospital admission and immunoglobulin costs.
Shabaninejad et al. (2017) [3]	Subcutaneous and intravenous administration of immunoglobulins in patients suffering from primary immunodeficiency in Iran.
Beauté et al. (2009) [4]	Subcutaneous and intravenous administration of immunoglobulins in patients suffering from primary immunodeficiency in France. IgGRT-costs ranged from €19,484 for home-based up to €25,583 for hospital-based intravenous IgGRT; costs of homebased subcutaneous IgGRT were estimated at €24,952 per year.
Simoens (2009) [5]	A pharmaco-economic expert review in primary immunodeficiency, comparing IgGRT regimens using different administration routes (intravenous or subcutaneous) and administration sites (hospital, home).
Sato et al. (2013) [6]	The average US healthcare cost per pneumonia episode was dependent on the in- or outpatient setting. The estimates were respectively $27,661 and $2,212 per episode. The observed frequencies of in- and out-patient pneumonia episodes were respectively 28% and 72%, with a mean duration of 10.2 days and 31.8 days. This results in a weighted average mean cost of nearly $9,338 per episode of pneumonia (US $ 2013).
McCain (2016) [7]	Autoimmune diseases. The total direct costs of rheumatoid arthritis were about $9 billion (US $ 2005) and it was estimated that about 1.5 million US citizens suffer from rheumatoid arthritis, yielding an annual healthcare cost of nearly $6,000 per patient. Similarly, the direct healthcare cost of psoriasis was estimated to be nearly $1,698 per patient.
Diel et al. (2018) [8]<br>De la Rosa et al. (2016) [9]	The cost of bronchiectasis in Germany revealed a direct healthcare cost of €18,635 per patient and a mortality rate of 26.4% after 3 years of follow-up. The annual healthcare cost estimate is about €6,212. In Spain, the mean annual cost per patient was €4,671.90, ranging from €2,993.30 in mild bronchiectasis to €9,998.90 in severe bronchiectasis.
American Cancer Society (2017) [10]<br>Luengo-Fernandez (2013) [11]<br>Wilking NE et al. (2016) [12]	Cancer. Roughly $87.8 billion was spent in 2014 in the US on cancer-related health care, equivalent to nearly $5,850 per patient affected by cancer. The cancer related healthcare cost

IACR data [13]	was estimated to be €51 billion in 2009 in Europe. According to Wilking, this cost increased from €79 to €86 billion during 2005-2014 (in 2014 prices) or nearly €8,430 per patient affected by cancer, based on the European Registry data.
NIHDI Belgium [14]	The annual IgGRT direct acquisition cost is set at €20,000; this should reflect the cost of a mixed intravenous/subcutaneous treatment population and the lower acquisition cost for some IgGRT products: e.g. annual acquisition cost in Belgium may be €15,814 or €16,452. The healthcare cost (GP visits, medicinal products) of minor infections is set at €75 per episode.

[1] Liu Z, Albon E, Hyde C. Department of Public Health and Epidemiology West Midlands Health Technology Assessment Group. The effectiveness and cost effectiveness of immunoglobulin replacement therapy for primary immunodeficiency and chronic lymphocytic leukaemia: a systematic review and economic evaluation. DPHE 2006, Report Number 54. University of Birmingham.

[2] Sadeghi B et al. Economic burden of common variable immunodeficiency: Annual cost of disease. Expert Review of Clinical Immunology (2015) 11(5):1-8.

[3] Shabaninejad H, Asgharzadeh A, Rezapour A, Rezaei N. Cost-effectiveness analysis of subcutaneous immunoglobulin replacement therapy in Iranian patients with primary immunodeficiencies. Med J Islam Repub Iran. 2017(17 Dec);31.94.

[4] Beauté J et al. Economic evaluation of immunoglobulin replacement in patients with primary antibody deficiencies. Clinical and Experimental Immunology 2009, 160: 240–245.

[5] Simoens S. Pharmacoeconomics of immunoglobulins in primary immunodeficiency. Expert Rev. Pharmacoeconomics Outcomes Res. 2009; 9(4), 375-386.

[6] Sato R et al. Community-Acquired Pneumonia Episode Costs by Age and Risk in Commercially Insured US Adults Aged >50 Years. Appl Health Econ Health Policy (2013) 11:251–258. DOI 10.1007/s40258-013-0026-0.

[7] McCain J. The Disease Burden of the Most Common Autoimmune Diseases. MANAGED CARE, July 2016.

[8] Diel R, Chalmers JD, Rabe KF, Nienhaus A, Loddenkemper R, Ringshausen FC. Economic burden of bronchiectasis in Germany. European Respiratory Journal 2018; DOI: 10.1183/13993003.02033-2018.

[9] De la Rosa D et al. Annual direct medical costs of bronchiectasis treatment: Impact of severity, exacerbations, chronic bronchial colonization and chronic obstructive pulmonary disease coexistence. Chronic Respiratory Disease. April 2016, I-II. DOI: 10.1177/1479972316643698.

[10] American Cancer Society. Cancer Action Network. The Costs of Cancer. 2017.

[11] Luengo-Fernandez R, Leal J, Gray A, Sullivan R. Economic burden of cancer across the European Union:a population-based cost analysis. Lancet Oncology 2013; 14: 1165–74.

[12] Wilking NE, Hofmarcher T, Lindgren P, Jönsson B. The burden and direct cost of cancer in Europe (EU-28). Journal of Clinical Oncology 2016 34:15_suppl, 6618-6618.

[13] The International Association of Cancer Registries (IACR) via the association's website (http://www.iacr.com.fr/). The Global Initiative for Cancer Registry Development (GICR), coordinated by IARC, via the GICR initiative's website (http://gicr.iarc.fr).

[14] NIHDI webtool on pricing of medicines, via https://ondpanon.riziv.fgov.be/SSPWebApplicationPublic/fr/Public/ProductSearch.
