# Supplementary material for: Immunoglobulin Replacement Therapy is critical and cost-effective in increasing life expectancy and quality of life in patients suffering from Common Variable Immunodeficiency Disorders (CVID): A health-economic assessment
Source: PLoS One. 2021 Mar 4;16(3):e0247941. doi: 10.1371/journal.pone.0247941 (PMC7932530; doi:10.1371/journal.pone.0247941)
Supplement: S3 Table — (PDF) [file pone.0247941.s003.pdf]

S3 Table. Matrix of transition probabilities between the different health states

Comparator

START \ END	asymp	infmin	infmaj	autoimm	CLD	cancer	dead	SUM
asymp	0.8753	0.0362	0.0128	0.0191	0.0419	0.0077	0.0068	1.0000
infmin	0.8616	0.0500	0.0128	0.0191	0.0419	0.0077	0.0068	1.0000
infmaj	0.6382	0.0362	0.2500	0.0191	0.0419	0.0077	0.0068	1.0000
autoimm	0.0000	0.0000	0.0128	0.9307	0.0419	0.0077	0.0068	1.0000
CLD	0.0000	0.0000	0.0000	0.0000	0.9854	0.0077	0.0068	1.0000
cancer	0.0000	0.0000	0.0000	0.0000	0.0000	0.9932	0.0068	1.0000
dead	0.0000	0.0000	0.0000	0.0000	0.0000	0.0000	1.0000	1.0000

Intervention

START \ END	asymp	infmin	infmaj	autoimm	CLD	cancer	dead	SUM
asymp	0.9069	0.0279	0.0049	0.0191	0.0323	0.0077	0.0010	1.0000
infmin	0.8963	0.0385	0.0049	0.0191	0.0323	0.0077	0.0010	1.0000
infmaj	0.8156	0.0279	0.0963	0.0191	0.0323	0.0077	0.0010	1.0000

autoimm	0.0000	0.0000	0.0049	0.9540	0.0323	0.0077	0.0010	1.0000
CLD	0.0000	0.0000	0.0000	0.0000	0.9913	0.0077	0.0010	1.0000
cancer	0.0000	0.0000	0.0000	0.0000	0.0000	0.9990	0.0010	1.0000
dead	0.0000	0.0000	0.0000	0.0000	0.0000	0.0000	1.0000	1.0000

The seven health states used in the model are shown. Abbreviations: asymp, controlled state; autoimm, autoimmunity; CLD, chronic lung disease (including bronchiectasis); infmin, minor infections; infmaj, major infections.
