# Supplementary material for: Immunoglobulin Replacement Therapy is critical and cost-effective in increasing life expectancy and quality of life in patients suffering from Common Variable Immunodeficiency Disorders (CVID): A health-economic assessment
Source: PLoS One. 2021 Mar 4;16(3):e0247941. doi: 10.1371/journal.pone.0247941 (PMC7932530; doi:10.1371/journal.pone.0247941)
Supplement: S4 Table — (PDF) [file pone.0247941.s004.pdf]

**S4 Table. Monte Carlo simulations and sensitivity analyses.**

| Distributional parameters for transition probabilities, utilities and costs | | | | | |
|---|---|---|---|---|---|
| *Health state* | *Type of distribution* | *Expected value E(x)* | *Variance V(x)* | *Parameters* | |
| | | | | $\alpha$ | $\beta$ |
| **Monthly transition probability – Intervention group (≥450mg/kg IgGRT)** | | | | | |
| Minor infection | β | 0.0279 | $3.10*10^{-6}$ | 244 | 8,505 |
| Major infection | β | 0.0049 | $0.15*10^{-6}$ | 159 | 31,906 |
| CLD | β | 0.0323 | $2.60*10^{-6}$ | 388 | 11,627 |
| Mortality | β | 0.0010 | $1.59*10^{-8}$ | 64 | 63,348 |
| **Monthly transition probability – Control group (≤100mg/kg IgG)** | | | | | |
| Minor infection | β | 0.0362 | $20.53*10^{-6}$ | 62 | 1,639 |
| Major infection | β | 0.0128 | $2.58*10^{-6}$ | 63 | 4,857 |
| CLD | β | 0.0419 | $27.41*10^{-6}$ | 61 | 1,402 |
| Mortality | β | 0.0068 | $0.73*10^{-6}$ | 64 | 9,218 |
| **Utility** | | | | | |
| Minor infection | β | 0.791 | 0.005241 | 24 | 6 |
| Major infection | β | 0.736 | 0.004213 | 33 | 12 |
| Chronic Lung Disease | β | 0.680 | 0.003444 | 42 | 20 |
| Auto-immune disease | β | 0.807 | 0.009032 | 13 | 3 |
| Cancer | β | 0.611 | 0.002213 | 65 | 41 |
| **Monthly costs** | | | | | |
| IgG | γ | 1,666.67 | 27,777.78 | 100 | 16.7 |
| Major infection | γ | 9,000.00 | 810,000.00 | 100 | 90.0 |
| Auto-immune disease | γ | 330.00 | 1,089.00 | 100 | 3.3 |
| Chronic Lung Disease | γ | 450.00 | 2,025.00 | 100 | 4.5 |
| Cancer | γ | 600.00 | 3,600.00 | 100 | 6.0 |

The cost estimates came from various geographic sources. Considering the regional diversity in cost estimates, we built a core health economic model, in which the published cost data were used and converted to actual value in Euro, rounded to the upper 100 Euro level. We believe more accurate estimates per health state are needed in country-specific analyses for country-specific use; but a country-specific health economic analysis would require checking the country-specific databases and tariffs which goes beyond the purpose of our study. We compensated the uncertainty in our estimates by performing sensitivity analyses on the efficiency impact of changing the cost estimates as follows:

| Results from Monte Carlo simulations on cost estimates | | | | | |
|---|---|---|---|---|---|
| | *IgGRT* | *Infection Major* | *Auto-immune disease* | *Chronic Lung Disease* | *Cancer* |
| Mean | 1.663 | 8.991 | 329 | 453 | 600 |
| Median | 1.656 | 8.976 | 328 | 452 | 599 |
| Minimum | 1.065 | 6.520 | 234 | 303 | 448 |
| Maximum | 2.227 | 12.079 | 453 | 584 | 818 |
