# Supplementary material for: Immunoglobulin Replacement Therapy is critical and cost-effective in increasing life expectancy and quality of life in patients suffering from Common Variable Immunodeficiency Disorders (CVID): A health-economic assessment
Source: PLoS One. 2021 Mar 4;16(3):e0247941. doi: 10.1371/journal.pone.0247941 (PMC7932530; doi:10.1371/journal.pone.0247941)
Supplement: S5 Table — (PDF) [file pone.0247941.s005.pdf]

S5 Table. Results from one-way sensitivity analyses

Analysis	ICER (€/LY)	ICUR (€/QALY)
3% decrease of future value for both costs and effects	€ 41,401.40	€ 65,640.25
Cohort baseline % changes in autoimmunity	No effect (< 1%)	No effect (< 1%)
Decrease by 10% the IgGRT costs	€ 26,836.00	€ 42,795.00
Mortality rate from ESID 2018 [1]	€ 28,135.00	€ 44,916.00
Annual rate of bronchiectasis (21.9%) instead of CLD	€ 29.229,75	€ 46.533,24
100% (vs 50%) effect size on bronchiectasis	€ 29.161,40	€ 46.125,66
Increased mortality rate (+ 50%) of bronchiectasis	€ 27.778,97	€ 43.876,01

Abbreviations: CLD, chronic lung disease; ESID, European Society for Immunodeficiencies; ICER, incremental cost-effectiveness ratio; ICUR, incremental cost-utility ratio; IgGRT, immunoglobulin replacement therapy; LY, life-years; QALY, quality-adjusted life-years.

[1] Odnoletkova et al. Orphanet Journal of Rare Diseases. The burden of common variable immunodeficiency disorders: a retrospective analysis of the European Society for Immunodeficiency (ESID) registry data. 13 (201): 2018.
